# Detection of the Influenza A(H1N1)pdm09 Virus Carrying the K-15E, P83S and Q293H Mutations in Patients Who Have Undergone Bone Marrow Transplant

**DOI:** 10.1371/journal.pone.0094822

**Published:** 2014-04-16

**Authors:** Milene Mesquita, Paola Resende, Andressa Marttorelli, Viviane Machado, Carolina Q. Sacramento, Natalia Fintelman-Rodrigues, Juliana L. Abrantes, Rita Tavares, Marcelo Schirmer, Marilda M. Siqueira, Thiago Moreno L. Souza

**Affiliations:** 1 Measles and Respiratory viruses Laboratory, WHO/NIC, Oswaldo Cruz Institute, Fiocruz, Rio de Janeiro, RJ, Brazil; 2 Center for Bone Marrow Transplantation (CEMO), National Cancer Institute (INCa), Rio de Janeiro, RJ, Brazil; Fondazione IRCCS Policlinico San Matteo, Italy

## Abstract

The 2009 pandemic influenza A(H1N1)pdm09 virus emerged and caused considerable morbidity and mortality in the third world, especially in Brazil. Although circulating strains of A(H1N1)pdm09 are A/California/04/2009-like (CA-04-like) viruses, various studies have suggested that some mutations in the viral hemagglutinin (HA) may be associated with enhanced severity and fatality. This phenomenon is particularly challenging for immunocompromised individuals, such as those who have undergone bone marrow transplant (BMT), because they are more likely to display worse clinical outcomes to influenza infection than non-immunocompromised individuals. We studied the clinical and viral aspects of post-BMT patients with confirmed A(H1N1)pdm09 diagnosis in the largest cancer hospital in Brazil. We found a viral strain with K-15E, P83S and Q293H polymorphisms in the HA, which is presumably more virulent, in these individuals. Despite that, these patients showed only mild symptoms of infection. Our findings complement the discovery of mild cases of infection with the A(H1N1)pdm09 virus with the K-15E, P83S and Q293H mutations in Brazil and oppose other studies that have linked these changes with increased disease severity. These results could be important for a better comprehension of the impact of the pandemic influenza in the context of BMT.

## Introduction

Influenza A(H1N1)pdm09 emerged in April 2009, reaching developing countries more dramatically [1]. In Brazil, where influenza surveillance is neglected, more than 2000 A(H1N1)pdm09-associated deaths occurred in 2009 [2]. Understanding the impact of A(H1N1)pdm09 is critically important for patients at higher risk of infection, such as immunocompromised individuals [3]–[7]. Cancer and transplant patients have atypical clinical presentations of influenza-like illness (ILI), which may delay adequate clinical interventions [8], leading to worse clinical outcomes [5], [9]–[12]. In Brazil, over 7% of A(H1N1)pdm09-related deaths occurred in immunocompromised individuals [2].

In addition to predisposing conditions in the host, specific polymorphisms in the viral genome could enhance viral virulence [13]–[18]. Although no major antigenic variations in the hemagglutinin (HA) of circulating strains of A(H1N1)pdm09 have been found, several polymorphic strains have been described, constituting seven clades [17], based concatenated viral genomes [17], [19], [20]. Sequences from the HA segment reproduce the phylogenetic topology for, the presumably more virulent, clade 6 and 7 viruses [17]. In South America, viruses from clades 5, 6 and 7 co-circulated [21]. Polymorphisms found in clade 6 strains have been identified and include the K-15E, P83S and Q293H mutations (ESH strain) [5], [13]–[15], [17]. Controversial interpretations of ESH strain-related morbidity and mortality [5], [13]–[15], [17] have been raised. Potdar et al. [17] found a positive association between clade 6 and 7 viruses and deaths. Glinsky et al. [13] stated that more than 40% of the individuals who died due to A(H1N1)pdm09 were infected by a variant carrying the Q293H mutation. However, the ESH strain has been found in some mild cases [14], [15], [20], reinforcing the fact that host-specific factors could also account for influenza-related clinical outcomes [22]. Considering that most of the information suggests a severe impact of ESH strains on the general population [13], it may be expected that this strain could be potentially life-threatening to immunocompromised individuals. We studied phylogenetically relevant changes in the HA of the A(H1N1)pdm09 virus in individuals that had undergone bone-marrow transplantation (BMT). Despite that, we found benign clinical outcomes, strengthening previous findings for the Brazilian population infected by the ESH strain [15].

## Material and Methods

### Ethics statement

Our study has been approved by the Ethics Committee (Comitê de Ética em Pesquisa; CEP; http://www.inca.gov.br/conteudo_view.asp?id=2380) at the Instituto Nacional de Câncer (INCa), Rio de Janeiro, Brazil. This institutional review board is led by Dr. Adriana Scheliga. Protocol was submitted under the number #18/2010 and the need for informed consent has been waived.

### Patients and data collections

Samples and clinical data from the 2009 pandemic from the National Reference Center for BMT (Centro de Transplante de Medula Óssea; CEMO) at the National Cancer Institute (INCa) in Rio de Janeiro, Brazil were sent to our laboratory as part of the influenza surveillance (Brazilian National Influenza Center/WHO). The minimal requirements for inclusion in this retrospective analysis of convenience samples were fever (>37.8°C), ILI and a previous history of BMT. From June to August 2009, nasopharyngeal swabs (NPAs) from 11 patients were collected. Of these patients, 9 had a confirmed diagnosis of A(H1N1)pdm09 (Table 1). Besides the cases under analysis, two control groups of patients were included. These were individuals with confirmed diagnosis of influenza A(H1N1)pdm09 and with date onset of illness from epidemiological weeks 24/2009 to 32/2010 (which represent the period of time from the mitigation phase of the pandemic to August 10^th^, 2010, when WHO issued recommendations for the post-pandemic period). As these samples were collected during the pandemics, numerous clinical-epidemiological forms were not entirely fulfilled. Nevertheless, we had 73 sequenced samples with completed clinical information, such as presence of comorbidities, immune status, presence of severe acute respiratory infection (SARI), requirement for hospitalization and clinical outcomes.

**Table 1:** Clinical- and viral-associated characteristics of patients who underwent BMT and had a confirmed diagnosis of A(H1N1)pdm09 infection.

Patient Number	Type of Cancer	Period of BMT	Sample Collection Date	Beginning of the Symptoms	Polymorphisms in Influenza A	Co-morbidity	Remission Cancer	Deceased
1	ALL	More than 6 months	15/Jul/09	14/Jul/09	K-15E, P83S and Q293H		No	No
2	AML	More than 6 months	23/Jul/09	23/Jul/09	K-15, P83S and Q293		No	29/Jun/10
3	CML	Within 6 months	24/Jul/09	18/Jul/09	K-15E, P83S and Q293R	GVHD	No	18/Jun/10
4	AML	More than 6 months	27/Jul/09	22/Jul/09	K-15E, P83S and Q293H		Yes	No
5	M	More than 6 months	29/Jul/09	27/Jul/09	K-15E, P83S and Q293H	Neutropenia	No	No
6	HL	Within 6 months	29/Jul/09	22/Jul/09	K-15E, P83S and Q293H	CLI	No	No
7	SM	More than 6 months	29/Jul/09	22/Jul/09	K-15E, P83S and Q293H		No	No
8	CML	More than 6 months	26/Jul/09	25/Jul/09	K-15E, P83S and Q293H		No	No
9	CML	More than 6 months	11/Aug/09	05/Aug/09	K-15E, P83S and Q293H		No	No

ALL – acute lymphoblastic leukemia, AML – acute myeloid leukemia, CML – chronic myelogenous leukemia, M – myelofibrosis, HL – Hodgkin's lymphoma, GVHD – graft-versus-host disease, CLI – chronic lung injury, NS – not sequenced.

### Sample collection and diagnosis

Nasopharyngeal Dacron swabs or aspirates (NPAs) were collected, and RNA was extracted using a viral RNA mini kit (Qiagen, CA), according to the manufacturer's instructions. RNA was eluted in 10 mM Tris-HCl, pH 8.0, with 1 mM EDTA (TE buffer) and stored at −70°C. This RNA was used for one-step Real-time RT-PCR assays for influenza subtyping according to the World Health Organisation (WHO) recommendations [23], [24]. Diagnosis for a range of other respiratory pathogens, such as coronavirus (229, 43 and 63), parainfluenza (1, 2, 3 and 4), human metapneumovirus, parechovirus, rhinovirus, RSV A/B, adenovirus and enterovirus, has been performed according to manufacturer's instructions (Fast Track Diagnosis, Luxemburg, Luxemburg).

### Cells and virus isolation

Madin-Darby canine kidney (MDCK) cells were cultured in Dulbecco's modified Eagle's medium (DMEM; GIBCO, Grand Island, NY) supplemented with 10% fetal bovine serum (FBS; Hyclone, Logan, Utah), 100 U/mL penicillin and 100 µg/mL streptomycin and were incubated at 37°C in 5% CO_2_ [23]. Virus isolation was performed in either 9-day-old embryonated eggs or in MDCK cells, as previously described [23]. We confirmed viral isolation using hemagglutination, neuraminidase activity or real-time RT-PCR assays [23]–[25]. Viruses were passaged no more than three times.

### Influenza HA sequencing

The influenza HA gene was sequenced by Sanger protocol, as described elsewhere [17], [24]. In Brief, RNA from patient's NPAs was extracted (viral RNA mini kit; Qiagen, CA) and subjected to one-step RT-PCR using Superscript III and Platinum Taq with previously described primers [23]. Amplicons were purified and sequenced by the Sanger method (Big Dye Terminator Cycle Sequencing Ready Reaction kit, Applied Biosystems, CA). Fragments were then analyzed using an automatic sequencer (ABI PRISMTM 3100-avant Genetic Analyzer; PE, Applied Biosystems). Consensus sequences for HA were generated in SeqEd (Applied Biosystems) and aligned to other sequences deposited in GenBank using the ClustalW algorithm in Megalign (Mega software 4.1). The products were analysed in an ABI Prism 3130XL genetic analyser (Life technologies). The dataset generated were assembled in Sequencher 5.0 software (GeneCodes Corporation, Michigan, USA) with a HA reference sequence, A/California/4/2009 (GenBank accession number: FJ966082). Of note, H1 numbering was used for HA throughout this study. Sequences were analyzed using neighbor-joining with bootstrap (1,000 times) and the Mega 4.1 software. Of note, H1 numbering was used for HA throughout this study. GenBank accession number for the sequences generated from this article are the following: KC967083-KC967090, CY052046-CY052050, CY052346-CY052350, CY054283, CY060444, CY060450, CY072074, CY072076, CY072082, CY072085-CY072088 and KJ417954-KJ417961. The amplified RT-PCR products were purified using the QIAquick PCR Purification kit (QIAGEN, Valencia, CA) and sequenced using a BigDye Terminator v3.1 Cycle Sequencing kit (Life technologies, CA).

### Statistical analysis

Standard descriptive statistics were used to describe the study population. Continuous variables were reported as the mean ± standard deviation or median (range) as appropriate. Comparative analyses between post-BMT patients and controls groups, non-imunocompromised individuals with mild outcome or otherwise healthy adults with severe/fatal outcomes were performed using OpenEpi software [26]. Significances were accessed through Fischer's exact test when *P* values <0.05. Odds ratio (OR) and 95%confidence intervals (CI) were registered when appropriate.

## Results

### Sampling and clinical data

The post-BMT patients in our study had a median age of 22 years (ranging from 9 to 40 years). Males accounted for 67% of the individuals. Patients who underwent BMT before or within 6 months of A(H1N1)pdm09 diagnosis were stratified because this time frame is critical for immunosuppression after BMT [27]. Two immunosuppressed patients, one with graft-versus-host disease (GVHD) and the other with chronic lung injury (CLI), received BMT 6 months prior to influenza diagnosis (Table 1). The other seven patients received BMT more than 6 months prior to influenza infection (Table 1). However, two patients in this last group were immunocompromised, due to remission of cancer or chronic neutropenia (<1.500 cells/mm^3^) (Table 1). Viruses infecting these patients were infectious, because they have been isolated in cell culture. Although we have searched for other respiratory viruses infection, all patients were negative (Fast-Track Diagnostic, Luxembourg, Luxembourg).

### Detection of mutant viruses and case-control analyses

**Figure 1:**
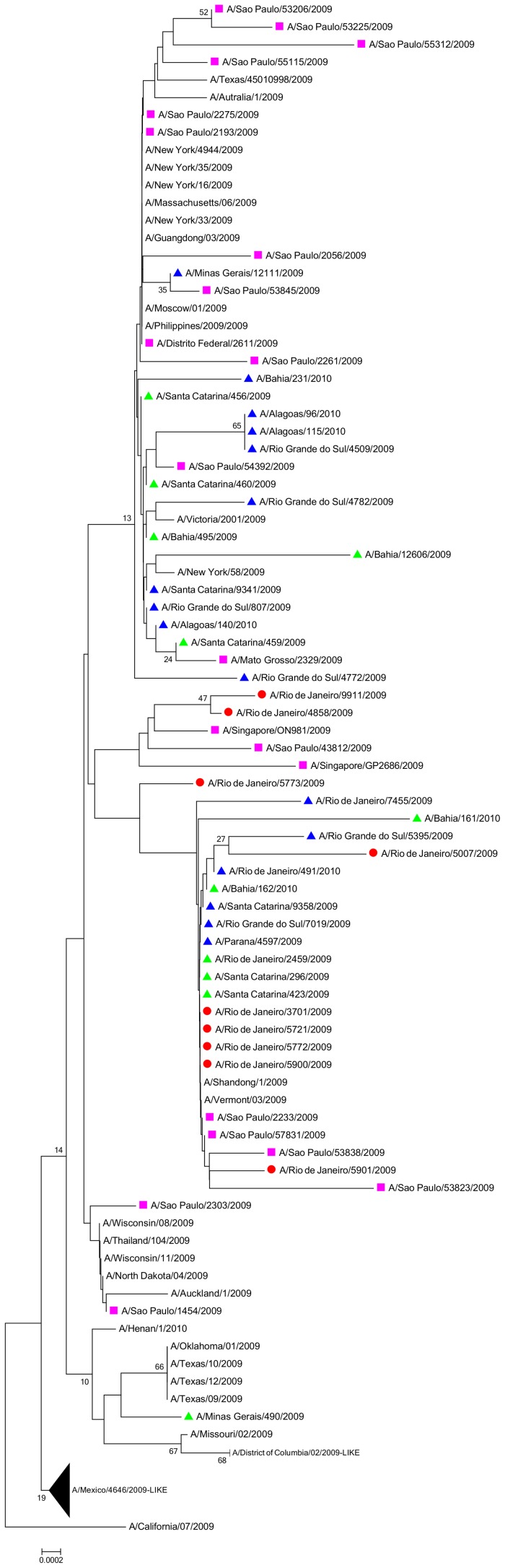
Phylogenetic tree of A(H1N1)pdm09 viruses sequenced from patients who underwent BMT. The scale bar indicates the number of amino acid changes per site. This tree is rooted by the HA sequence from the California/04/2009 strain. Bootstrap values below 10 are hidden. Influenza A(H1N1)pdm09 HA sequences are highlighted: post-BMT patients (red circles), Lee's work [15] (pink square), non-immunocompromised individuals with mild infection (green triangle) and otherwise healthy individuals with severe/fatal outcomes (blue triangle).

The A(H1N1)pdm09 HA was sequenced from all clinical samples. In eight cases, mutant viruses were found. The ESH strain was found in 7 patients, while one sample had the K-15E, P83S and Q293R mutations (Figure 1). In another patient, a strain with wild-type (WT) residues K-15 and Q293, and mutant P83S, was detected (KSQ strain; patient 2 in Table 1). The predisposing conditions of the patients indicate that they should be more likely to have worse clinical outcomes. Additionally, confirmed diagnosis with a presumably more virulent strain of A(H1N1)pdm09 could be even more critical for these individuals. Despite that, these patients displayed mild symptoms of infection, and hospitalization was not required. Comparisons of the influenza A(H1N1)pdm09 HA sequences from post-BMT patients with those from Lee et al [15], and other control groups (otherwise healthy individuals with severe/fatal outcomes or non-immunocompromised individuals with mild infection) are shown (Figure 1). As we can see, there is no specific pattern to cluster sequences together (Figure 1). In fact, this phylogenetic tree has low bootstrap values for the different branches (Figure 1); suggesting that independently of the clinical outcomes and/or predisposing condition, the different virus strains from this period looked alike.

Next, we compared the exposure to the virus carrying the three polymorphisms, ESH strain, as a risk factor to lead to different outcomes in post-BMT individuals and other control groups of patients. Of note, another required control would be a viral strain without the three polymorphisms. In our dataset, viruses genetically closer to the A/California/04/2009, also carry the change P83S. Mutation P83S is found in influenza A(H1N1)pdm09 viruses from clades 6 and 7 [17], and in Brazilian strains from the mitigation phase of the pandemics and afterwards. Since mutations K-15E and Q293H are the main ones associated with increased influenza virulence [13], [17], comparisons between ESH and KSQ strains correlation with disease severity could still be feasible. By selecting Brazilian samples from the pandemic period from our dataset, we found 73 sequenced samples from potential control groups. Among these, 21 cases were from healthy individuals, with registered information about absence of any comorbidity, and 51 cases were from non-immunocompromised individuals. Among healthy individuals, 3 had mild infection (no ESH virus detected), 13 had severe infection (5 ESH viruses) and 5 deceased (1 ESH virus). Out of the non-immunocompromised individuals, 11 had mild outcome (5 ESH viruses), 22 had severe infection (7 ESH viruses) and 18 deceased (7 ESH viruses). In Table 2, we compared ESH as a risk factor, over the KSQ strain, for post-BMT patients and other groups. Although ESH is presumably more lethal, the mild clinical outcome is more likely to occur in post-BMT patients than severe or fatal outcomes in otherwise healthy individuals (OR = 14.32, 95% CI 1.4–767.3; *P*<0.05) (Table 2, upper half). When the exposure to ESH is analyzed by comparing two groups with mild outcomes, post-BMT vs. non-immunocompromised patients, no significant difference is observed (Table 2, lower half). Despite that, there is a greater tendency of ESH-infected post-BMT patients progress to mild clinical infection than non-immunocompromised individuals (OR = 8.53 and 95% CI 0.71–495.4). Altogether, data from phylogenetic tree and contingency table, suggest that ESH strain correlation with severity or fatality may be questionable.

**Table 2:** Comparisons between post-BMT patients and controls groups with respect to strains of influenza A(H1N1)pdm09.

	Groups of patients and their respective outcome		
**Influenza A(H1N1)pdm09 strains (residues -15, 83 and 293)**	**Post-BMT with mild outcome**	**healthy individuals with severe or fatal outcomes**	* ***P*** ** values**
ESH	8	6	0.0088
KSQ	1	12	
**Influenza A(H1N1)pdm09 strains (residues -15, 83 and 293)**	**Post-BMT with mild outcome**	**non-immunocompromised with mild otcome**	* ***P*** ** values**
ESH	8	5	0.058
KSQ	1	6	

*P values were determined by Fischer's exact test, values below 0.05 were considered to be statistically significant.

Ours findings oppose the previously observed impact of the ESH strain on the general population [13], [15], [17]. Our patients were easily distinguishable in terms of their specific genetic backgrounds, ethnicity and previous history of treatments and clinical interventions. Therefore, it seems unlikely that an impaired virus replication could have occurred as a consequence of any specific cancer- or BMT-related interventions. Although the works on influenza infection in post-BMT patients is, in general, limited by the small number of patients (around a dozen individuals) [28], contributions such as this are important for further meta-analysis investigations – which may strengthen the isolated findings displayed in the literature.

## Discussion

The association of clinical data and viral sequences is important for the identification of novel virulence markers [22]. Although such an approach has been used for surveillance, it takes the host's genetic background for granted. We believe that surveillance focused on immunocompromised patients could, in many cases, yield more pragmatic insights for public health concerns. Because these post-BMT patients tend to have worse clinical outcomes due to influenza infection, the identification of mild symptoms for a presumably more virulent virus could mean that enhanced pathogenicity of this variant is questionable or that some specific factor associated with this population may impair virus infection/replication.

With respect to the ESH strain, our findings complement the studies by Graham et al. and Lee et al. [14], [15] and oppose those by Glinsky et al. and Potdar et al. [13], [17]. ESH strain may be included into the clade 6 of influenza A(H1N1)pdm09, which has been associated with morbidity and mortality [17]. Nevertheless, clade-6 viruses possess mutations in genes other than HA, which could naturally influence its virulence. Glinsky et al [13] found an exceedingly high prevalence of Q293H mutation in individuals that deceased. Based solely on this last work, association between Q293H and mortality would be even more significant than the one presented by the change D222G in viral HA [16], [18], [29], [30]. It should be taken into account that, ESH strain may have simply emerged during the peak of pandemic influenza circulation [15], [31]. As so, ESH strain has been detected in mild cases of 2009 pandemic virus infection, by us and others in different countries [15], [31]. By studying Brazilian samples from the same period, Lee and colleagues [15] suggested that rather than real phenotypic changes that could increase influenza A(H1N1)pdm09 virulence, the ESH strain was emerged as a stochastic event during the peak of virus circulation during the Brazilian winter. Therefore, we agree with this interpretation that increased mortality and hospitalizations in Brazil during the epidemiological weeks 31 to 35 of 2009 were due to enhanced influenza circulation through predisposed hosts than to the emergence of the ESH strain. As it has been demonstrated, major event that led to 2009 influenza morbidity and mortality, including in otherwise healthy individuals, was the susceptible of the human population to influenza viruses with very low glycosylated HA, such as influenza A(H1N1)pdm09 [32], [33]. Nevertheless, accumulation of global data from different cohorts/groups of patients is needed to reach more decisive conclusions on the importance of these mutations.

Finally, our results could stimulate a debate among investigators working on the molecular epidemiology/surveillance of influenza about critical mutations in the A(H1N1)pdm09 strain and could be interesting for physicians working on BMT.
